# Oxy210, a novel inhibitor of hedgehog and TGF‐β signalling, ameliorates hepatic fibrosis and hypercholesterolemia in mice

**DOI:** 10.1002/edm2.296

**Published:** 2021-08-31

**Authors:** Simon T. Hui, Feng Wang, Frank Stappenbeck, Samuel W. French, Clara E. Magyar, Farhad Parhami, Aldons J. Lusis

**Affiliations:** ^1^ Department of Medicine Division of Cardiology David Geffen School of Medicine University of California Los Angeles California USA; ^2^ MAX BioPharma, Inc Santa Monica California USA; ^3^ Department of Pathology & Laboratory Medicine David Geffen School of Medicine University of California Los Angeles California USA

**Keywords:** cholesterol metabolism, liver fibrosis, non‐alcoholic Steatohepatitis, oxysterol, therapeutics

## Abstract

**Aims:**

Non‐alcoholic steatohepatitis (NASH) is associated with increased overall morbidity and mortality in non‐alcoholic fatty liver disease (NAFLD) patients. Liver fibrosis is the strongest prognostic factor for clinical outcomes, liver‐related mortality and liver transplantation. Currently, no single therapy or medication for NASH has been approved by the U.S. Food and Drug Administration (FDA). Oxy210, an oxysterol derivative, displays the unique property of antagonizing both Hedgehog (Hh) and transforming growth factor‐beta (TGF‐β) signalling in primary human hepatic stellate cells (HSC). We hypothesized that inhibition of both Hh and TGF‐β signalling by Oxy210 could reduce hepatic fibrosis in NASH. In this study, we examined the therapeutic potential of Oxy210 on NASH in vivo.

**Methods:**

We examined the effect of Oxy210 treatment on Hh and TGF‐β pathways in HSC. The efficacy of Oxy210 on liver fibrosis was tested in a ‘humanized’ hyperlipidemic mouse model of NASH that has high relevance to human pathology.

**Approach and Results:**

We show that Oxy210 inhibits both Hh and TGF‐β pathways in human HSC and attenuates baseline and TGF‐β‐induced expression of pro‐fibrotic genes *in vitro*. Oral delivery of Oxy210 in food resulted in significant liver exposure and significantly reduced hepatic fibrosis in mice over the course of the 16‐week study with no apparent safety issues. Additionally, we observed several benefits related to NASH phenotype: (a) reduced plasma pro‐inflammatory cytokine and the corresponding hepatic gene expression; (b) reduced pro‐fibrotic cytokine and inflammasome gene expression in the liver; (c) reduced apoptosis in the liver; (d) reduced hepatic unesterified cholesterol accumulation; and (e) reduced plasma total and unesterified cholesterol levels.

**Conclusions:**

Oxy210 effectively ameliorated hepatic fibrosis and inflammation and improved hypercholesterolemia in mice. Our findings suggest that Oxy210 and related analogues are a new class of drug candidates that may serve as potential therapeutics candidates for NASH.

## Graphical abstract

Oxy210, an oxysterol derivative, displays the unique property of antagonizing both hedgehog (Hh) and transforming growth factor‐beta (TGF‐β) signalling in primary human liver stellate cells. Oxy210 effectively ameliorated hepatic fibrosis and inflammation and improved hypercholesterolemia in mice. Our findings suggest that oxysterol derivatives, such as Oxy210, are a new class of drug candidates to be considered for NASH therapy.

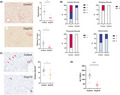

## 1 INTRODUCTION

With the increasing prevalence of obesity, diabetes and metabolic syndrome, non‐alcoholic fatty liver disease (NAFLD) has rapidly become the most common form of chronic liver disease worldwide and the top non‐viral cause of liver failure requiring liver transplantation.^29^ NAFLD comprises of a spectrum of hepatic abnormalities ranging from simple steatosis, inflammation, fibrosis and cirrhosis. Advanced NAFLD can eventually progress to end‐stage liver disease with elevated risk of hepatocellular carcinoma (HCC).^17^ While simple steatosis appears to be benign in most cases, the more advanced forms (known as non‐alcoholic steatohepatitis or NASH) are associated with increased overall morbidity and mortality. NASH is currently the second leading cause of cirrhosis and the leading cause of liver transplantation in patients over 55 years old.^36^ NAFLD patients have significantly increased all‐cause mortality, risk of cardiovascular disease, hepatocellular carcinoma and cirrhosis.^8^ The presence of fibrosis was found to be the strongest prognostic factor for clinical outcomes, liver‐related mortality and liver transplantation^1^, ^11^. Despite the increasing prevalence of NAFLD, no single therapy or medication for NASH has been approved by the U.S. Food and Drug Administration (FDA). Among numerous clinical candidates, agonists of the Farnesoid X receptor (FXR), such as the bile acid derivative, obeticholic acid and non‐steroidal agents, such as Tropifexor, are in advanced clinical trials for NASH.^28^ ^,^ ^35^ Even as these drugs near FDA approval, the complex nature of the disease leaves a sizable NASH patient population with a prevailing unmet need for new and improved NASH therapies to be dosed as single agents or combination therapies.

Fibrosis occurs due to uncontrolled wound healing in response to acute or chronic injuries.^27^ ^,^ ^31^ While many cellular and molecular mechanisms underlying tissue fibrosis have been identified, factors governing the progression from ectopic steatosis to NASH and fibrosis are largely unknown. A number of cell types in the liver microenvironment have been shown to contribute to the fibrotic process. The main contribution to this pathology is by myofibroblasts and hepatic stellate cells (HSC) that upon activation by a number of stimulants express pro‐fibrotic factors.^12^, ^31^ The hedgehog (Hh) pathway and especially the transforming growth factor‐beta (TGF‐β) pathway are among major signalling pathways that have been identified in activation of myofibroblasts and HSC ^6^ ^,^ ^19^ Increased amounts of Hh ligands and TGF‐β proteins have been detected in diseased liver, and inhibition of these pathways using specific antagonists or antibodies have been shown to ameliorate fibrotic process.^10^ ^,^ ^13^ ^,^ ^18^

**FIGURE 1:**
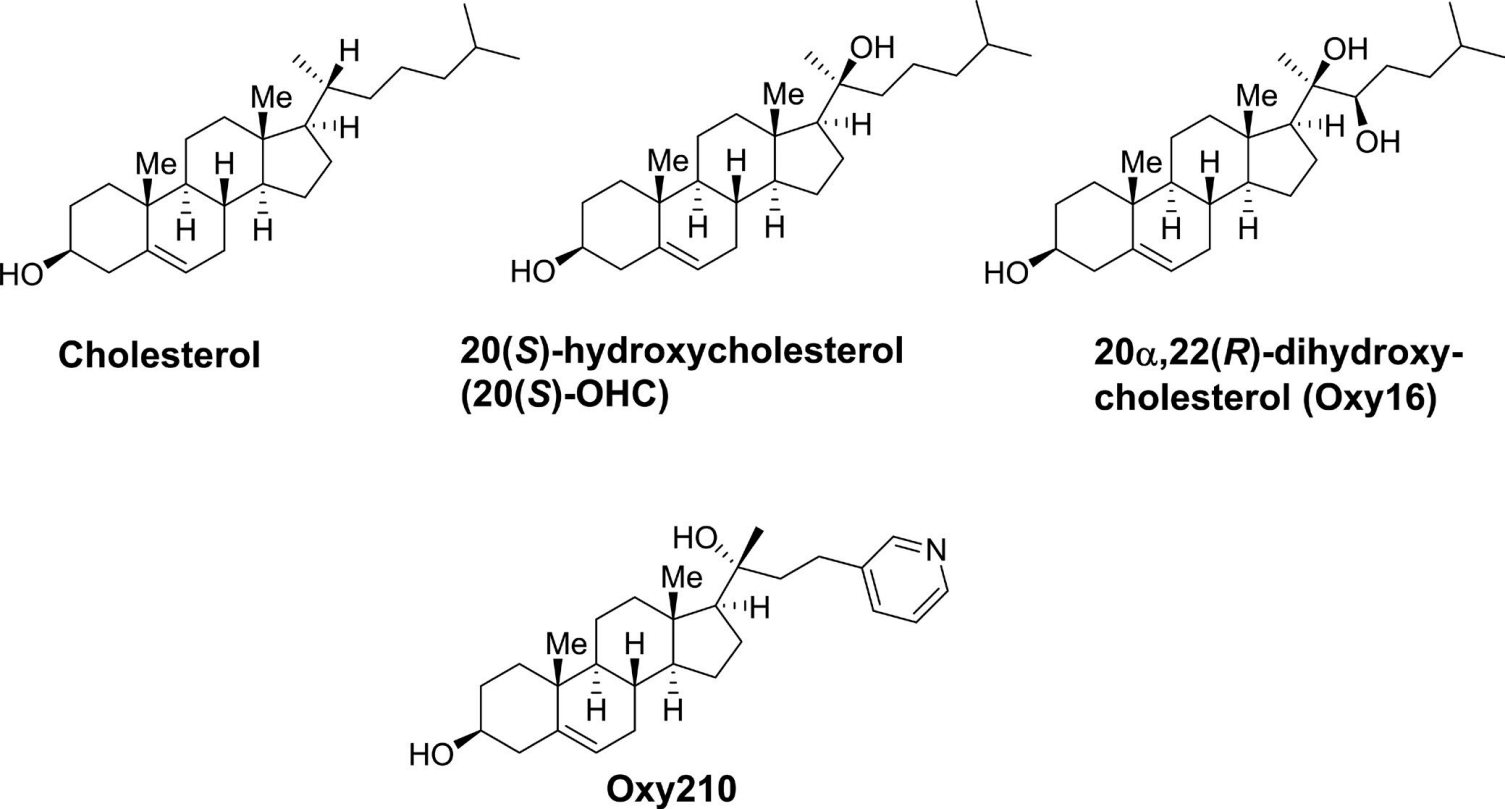
Chemical structures of cholesterol, oxysterol derivatives and Oxy210

Dwyer et al.^7^ first discovered, more than a decade ago, that certain naturally occurring oxysterols, such as 20(S)‐hydroxycholesterol and 20α,22(*R*)‐dihydroxy‐cholesterol (Oxy16), two closely related known metabolites in the biosynthesis of steroid hormones, can modulate Hh signalling in mesenchymal cells (Figure 1). 20(*S*)‐hydroxycholesterol [20(*S*)‐OHC] activates Hh signalling through allosteric activation of the transmembrane protein, Smoothened (SMO).^24^ By contrast, Oxy16 acts as a Hh pathway antagonist but does not display SMO binding.^24^, ^32^ Through structure‐activity relationship studies, we have gained a detailed understanding of how the molecular structure of the oxysterol can determine its activity as an agonist or antagonist of Hh pathway signalling. MAX BioPharma has developed a series of oxysterol derivatives targeting the Hh pathway for cancer therapy. One such derivative, Oxy210, displays the unique property of antagonizing both Hh and TGF‐β signalling in fibroblasts, in tumour cells,^30^ and in primary human HSC. Given the reported role of these two pathways in liver fibrosis and hepatic carcinoma, we anticipated that Oxy210 may inhibit liver pathology by its dual inhibitory effects on Hh and TGF‐β signalling pathways. We hypothesized that inhibition of both Hh and TGF‐β signalling by Oxy210 could reduce hepatic fibrosis in NASH. Dual inhibition of these pathways in the context of NASH is a novel concept and that has not been previously investigated. This prompted the current study, in which we examine the therapeutic potential of Oxy210 on NASH in mice.

In this study, we investigated the therapeutic potential of Oxy210 on NASH in mice. To date, many mouse models (both genetic and diet‐induced) have been developed to represent the pathophysiology, morphological findings, biochemical changes and clinical features of human NAFLD/NASH, but none offer a satisfactory system for studying the progression of NASH. The commonly used methionine‐choline‐deficient diet (MCDD) induces inflammation and fibrosis but does not exacerbate obesity and insulin resistance associated with NASH in humans.^16^ Mice fed with high‐fat or high‐fructose diets exhibit metabolic syndrome and hepatic steatosis, but progression to NASH and fibrosis is rare. The over‐nutrition models using the Amylin liver non‐alcoholic steatohepatitis (AMLYN) or Gubra Amylin NASH (GAN) diet produce relevant metabolic and histological features of human NASH.^3^, ^4^ However, the time required for NASH development is relatively long (30 weeks).^3^, ^4^ We recently employed several innovative approaches to develop a mouse disease model that recapitulates human NASH in shorter timeframe (16 weeks).^14^ Comparison of our mouse transcriptomic network with that of human NASH patients showed significant overlaps and resemblance between the two species.^14^ We show that Oxy210 inhibits both Hh and TGF‐β pathways in human HSC and attenuates TGF‐β‐induced expression of pro‐fibrotic genes in vitro. Furthermore, using this ‘humanized’ hyperlipidemic mouse model of NASH,^14^ we demonstrate that oral administration of Oxy210 ameliorates liver fibrosis and reduces inflammation, and lipid deposition. Our findings suggest that Oxy210 and related analogues may serve as potential therapeutic candidates for NASH.

## 2 MATERIALS AND METHODS

### 2.1 Animal studies

The breeding and characterization of transgenic mice expressing human cholesteryl ester transfer protein (CETP) and the human APOE*3‐Leiden (E3L) were described previously.^2^, ^14^ To generate mice for the fibrosis studies, male C57BL/6J mice carrying both transgenes were bred to BXD19/TyJ females. F1 progeny carrying both transgenes were used for the studies. Animals were maintained on a 12 h light‐dark cycle with *ad libitum* access to food and water. Control mice were fed a ‘Western’ diet (WD, 33 kcal % fat from cocoa butter and 1% cholesterol, Research Diets, cat# D10042101) whereas Oxy210‐treated mice were fed the same diet with supplementation of Oxy210 at 4 mg/g or 0.4% (w/w). Based on an average consumption of 3 grams of food by a 30 gram mouse per day, this formulation of Oxy210 in food amounts to a dose of 400 mg/kg/day. However, based on actual average consumption of food during the study, the *bona fide* dose delivered was about 260 mg/kg/day. Synthesis and characterization of Oxy210 were previously described by MAX BioPharma.^30^ Body composition was measured by NMR (Bruker Biospin Corp). Euthanasia was carried out with isoflurane vapour followed by cervical dislocation. All animal work was approved by the UCLA Animal Research Committee, the IACUC.

### 2.2 Cell culture and reagents

The primary human HSC were obtained from Zen‐Bio (Research Triangle Park) and LX‐2 human HSC cell line was obtained from Millipore Sigma. All cells were cultured in DMEM containing 10% FBS and antibiotics penicillin and streptomycin. TGF‐β1 was obtained from R&D Systems. Primary human HSC were treated in DMEM containing 0.1% FBS overnight and then pretreated for 2 h with Oxy210 or vehicle control. The cells were then treated with TGF‐β (10 ng/ml) in the absence or presence of Oxy210. RNA was extracted after 24 h and gene expression was analysed by qPCR and normalized to GAPDH expression. For assessment of Hh responsiveness, primary human HSC were pretreated for 2 h with Oxy210 as indicated in DMEM containing 5% FBS. The cells were then treated with conditioned medium from human pancreatic tumour cells, CAPAN‐1 cells (CM) in the absence or presence of Oxy210.^32^ CM was previously reported to contain biologically active Shh and Ihh.^32^ After 72 h, RNA was extracted and gene expression was analysed by qPCR and normalized to GAPDH expression. For cell proliferation assays, primary human HSC or LX‐2 cells were cultured in growth medium and treatment with Oxy210 was performed in DMEM containing 1% FBS in 12‐well plates at 20% confluence for 5 days and then trypsinized, spun down and resuspended in fresh medium. An aliquot of cell suspension was applied to a hemocytometer, and the cells were counted under a light microscope as previously described.^30^ Oxy210 was prepared and characterized by MAX BioPharma according to our published reports.^30^

### 2.3 RNA isolation and quantitative PCR

Total RNA was extracted from the cells or livers of the mice with the RNeasy Plus Mini Kit from Qiagen according to the manufacturer's instructions. One microgram of RNA was reverse‐transcribed using an iScript Reverse Transcription Supermix from Bio‐Rad, to make single‐stranded cDNA. The cDNAs were then mixed with Qi SYBR Green Supermix (Bio‐Rad) for quantitative RT‐PCR assay using a Bio‐Rad I‐cycler IQ quantitative thermocycler. All PCR samples were prepared in triplicate wells in a 96‐well plate. After 40 cycles of PCR, melt curves were examined in order to ensure primer specificity. Fold changes in gene expression were calculated using the ΔΔCt method. Sequence of primers used is listed in Table S1.

### 2.4 Plasma lipids, cytokines and metabolites

Mice were fasted for 4 h. Plasma glucose and lipids were measured by colorimetric analysis as described.^15^ Plasma insulin was measured using the mouse insulin ELISA kit (80‐INSMS‐E01) from Alpco.^2^ Quantification of plasma cytokines was carried out by electro‐chemiluminescence (ECLIA)‐based multiplex assay using U‐PLEX 3‐plex panel for IL‐6, MCP‐1 (CCL2) and TNFα (Meso Scale Discovery) as per manufacturer's instructions. Plates were measured using the MSD QuickPlex SQ120, and the cytokine concentrations were calculated from standard curves using Discovery Workbench 4.012 software.

### 2.5 Plasma and liver exposure in mice consuming Oxy210 formulated in Western Diet (WD)

Ten male C57BL/6J mice were fed Oxy210 formulated in WD chow *ad libitum* for 96 h. All mice in this study were monitored for clinical signs, body weights (g) and food consumption (g/mouse/day). At 96 h, terminal blood and liver samples were collected and the concentration of Oxy210 determined in serum and liver tissue based on our published bioanalytical methods.^30^

### 2.6 Quantitative assessment of steatosis and fibrosis in the liver

Liver lipids were extracted and quantitated as described.^15^ For histological examination and fibrosis quantitation, livers were fixed in 10% formalin, embedded in paraffin, sectioned at 5 µm and stained with picrosirius red. Slides were scanned at 20x magnification (Aperio ScanScope XT, Leica Biosystems). Using Definiens Tissue Studio (Definiens AG), we designed a fully automated image analysis algorithm that quantifies fibrosis as the percentage area of the whole tissue section, excluding normal vascular wall and liver capsular collagen. This method was previously validated by showing that the percentage of fibrosis measured strongly correlated with blinded assessments by liver pathologist and collagen content as measured by hydroxyproline assays.^14^ Pathological fibrosis in the entire liver cross‐section was quantified and expressed as percentage area over total area of the section.

### 2.7 Assessment of apoptosis by immunohistochemical assay

Paraffin‐embedded sections were cut at 4 μm thickness and paraffin removed with xylene and rehydrated through graded ethanol. Endogenous peroxidase activity was blocked with 3% hydrogen peroxide in methanol for 10 min. Heat‐induced antigen retrieval (HIER) was carried out for all sections in 0.001 M EDTA buffer, pH = 8.00 using a Biocare decloaker at 95℃ for 25 min. The slides were then stained with antibody against Cleaved caspase3 (Cell Signaling, cat#9661). The signal was detected using the DAKO EnVision+System and labelled polymer conjugated HRP anti‐rabbit secondary antibody (Agilent). All sections were visualized with the diaminobenzidine reaction and counterstained with haematoxylin. Positive stained cells were quantified by Definiens Tissue Studio software (Definiens AG).

### 2.8 Statistical analysis

Differences between two groups were compared using the Welch's t test. *p* values <0.05 were considered significant.

## 3 RESULTS

### 3.1 Oxy210 inhibits pro‐fibrotic gene expression and proliferation of human HSC

**FIGURE 2:**
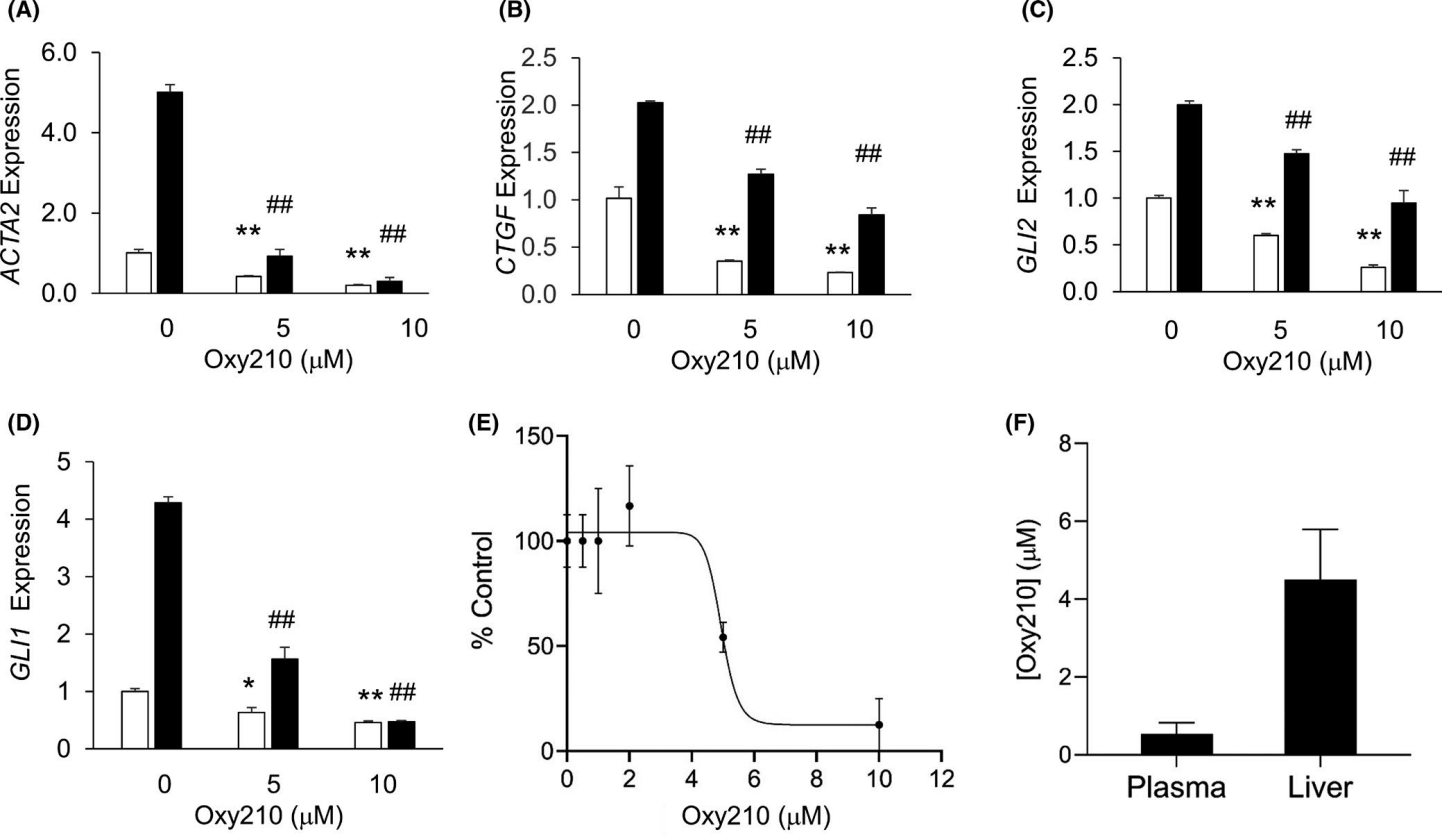
Effects of Oxy210 on primary human HSC and drug distribution in mice. (A‐C) Primary human HSC were cultured in DMEM containing 0.1% FBS overnight and then pretreated with 5 or 10 µM Oxy210 as indicated for 2 h. The cells were then treated with TGF‐β (10 ng/ml) (filled bars) or vehicle control (empty bars). Expression of *ACTA2* (A), *CTGF* (B) and *GLI2* (C) was analysed by qPCR and normalized to GAPDH expression. (D) Primary human HSC were pretreated for 2 h with Oxy210 as indicated in DMEM containing 5% FBS. The cells were then treated with conditioned medium from Capan‐1 cells (CM) (filled bars) or vehicle control (empty bars). After 72 h, RNA was extracted and expression of *GLI1* was measured by qPCR Data are presented as mean ±SD from 3 replicates per group. ##*p *< 0.01 vs. TGF‐β‐treated control; *p < 0.05 vs TGF‐β‐untreated control; **p < 0.01 vs TGF‐β‐untreated control. (E) Primary human HSC were plated at 10% confluence and treated with Oxy210 at the concentrations indicated for 5 days. Cells were then trypsinized and the number of cells determined. Data are reported as the mean of triplicate per group ±SD . (F) C57BL/6 mice were fed Oxy210 formulated in WD chow *ad libitum* for 96 h. At 96 h, terminal blood and liver samples were collected and the concentration of Oxy210 determined by LC/MS‐MS in serum and liver tissue. Data are presented as mean ± SD from 10 animals per group

Previously, we reported that Oxy210 inhibits Hh and TGF‐β signalling in mouse fibroblastic cells and non‐small lung cancer cells.^30^ As activation of Hh and TGF‐β pathways in HSC plays an important role in liver fibrosis during NASH progression, we examined whether Oxy210 inhibits these pathways in primary human HSC. Consistent with previous findings in other cell lines, when cultured primary human HSC were treated with 5 µM or 10 µM Oxy210, both basal and TGF‐β induced expression of pro‐fibrotic genes (*ACTA2* and *CTGF*) were significantly reduced (Figure 2A,B). Treatment with Oxy210 also decreased the basal as well as TGF‐β induced expression of Hh signalling mediator *GLI2* (Figure 2C), suggesting that Oxy210 inhibits TGF‐β induced non‐canonical Hh signalling in addition to its previously reported inhibitory effects on canonical Hh signalling. Furthermore, since non‐canonical TGF‐β signalling includes the activation of Hh signalling, these data suggest that at least some non‐canonical TGF‐β signalling may be inhibited by Oxy210. To assess the effects of Oxy210 on Hh signalling, we treated primary human HSC with CAPAN‐1 (a human pancreatic tumour cell line) conditioned medium, which we have previously reported to contain Hh ligands.^32^ Expression of *GLI1* was significantly decreased in Oxy210‐treated cells, in both basal and Hh ligand stimulated states (Figure 2D), suggesting that Oxy210 can effectively inhibit Hh canonical signalling in HSC. We also examined the effects of Oxy210 on HSC proliferation. Indeed, HSC proliferation was diminished in the presence of Oxy210, with a 46% decrease at the concentration of 5 µM (Figure 2E). The findings of reduction in TGF‐β‐induced expression of pro‐fibrotic genes by Oxy210 were also recapitulated in LX‐2 cells, a human hepatic stellate cell line (Figure S1A,B). The proliferation of LX‐2 cells was decreased by 94% at the concentration of 5 µM Oxy210 (Figure S1C).

### 3.2 Attenuation of hepatic fibrosis in mice by Oxy210

**FIGURE 3:**
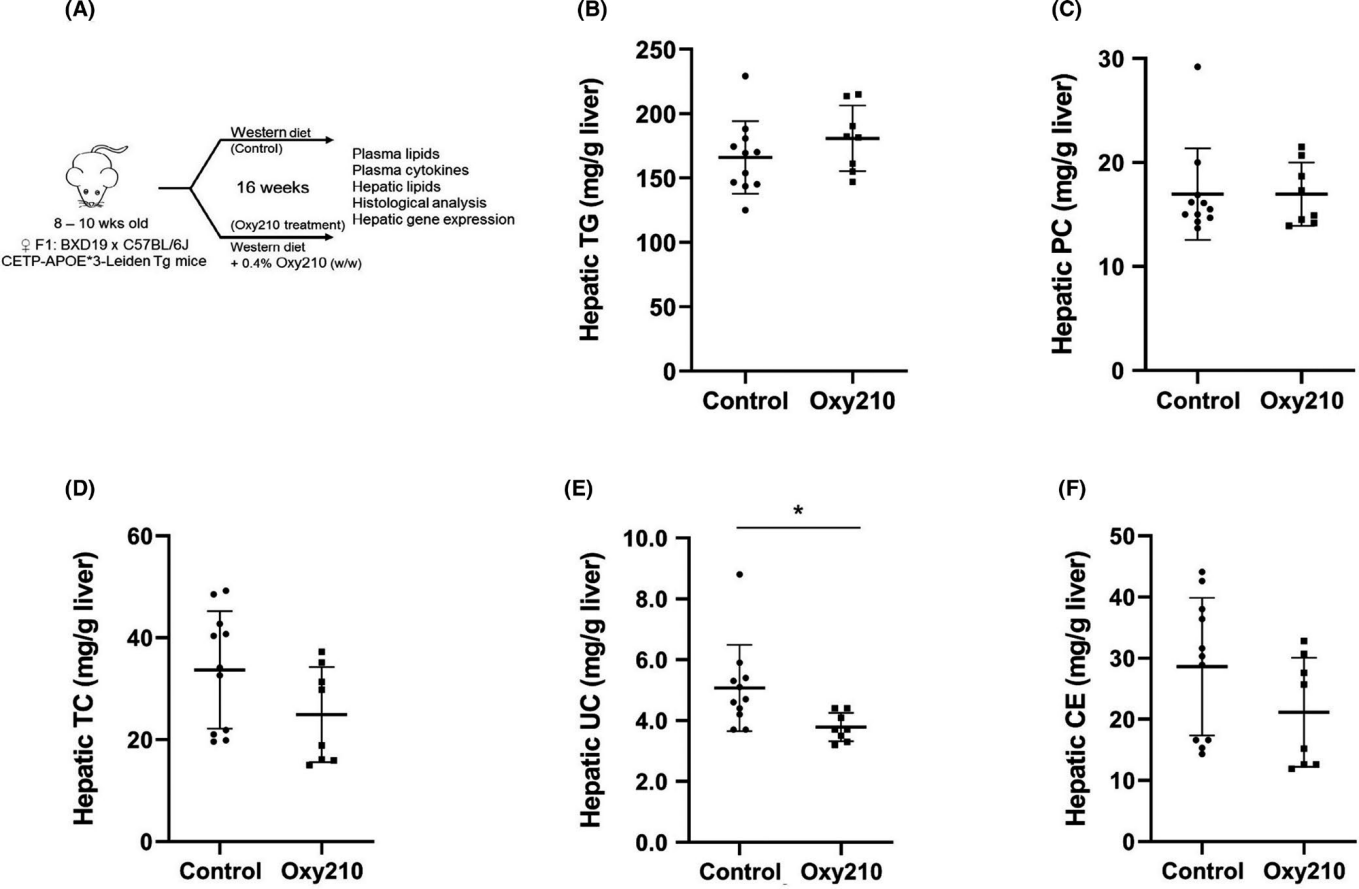
Changes in hepatic lipid levels by Oxy210 treatment. (A) Study design. Female mice carrying human APOE*3‐Leiden and CETP transgenes were fed *ad libitum* a ‘Western’ high‐fat, high‐cholesterol diet supplemented with Oxy210 (4 mg/g) for 16 weeks. Control mice were fed the same diet without Oxy210 supplementation. (B‐F) Hepatic levels of triglyceride (B), phosphatidylcholine (C), total cholesterol (D), unesterified cholesterol (E) and cholesterol ester (F) are presented as mean ± SD from 8 to 10 mice per group. Abbreviations: CE, cholesterol ester; PC, phosphatidylcholine; TC, total cholesterol; TG, triglyceride; UC, unesterified cholesterol. *denotes *p* < 0.05 versus control

**FIGURE 4:**
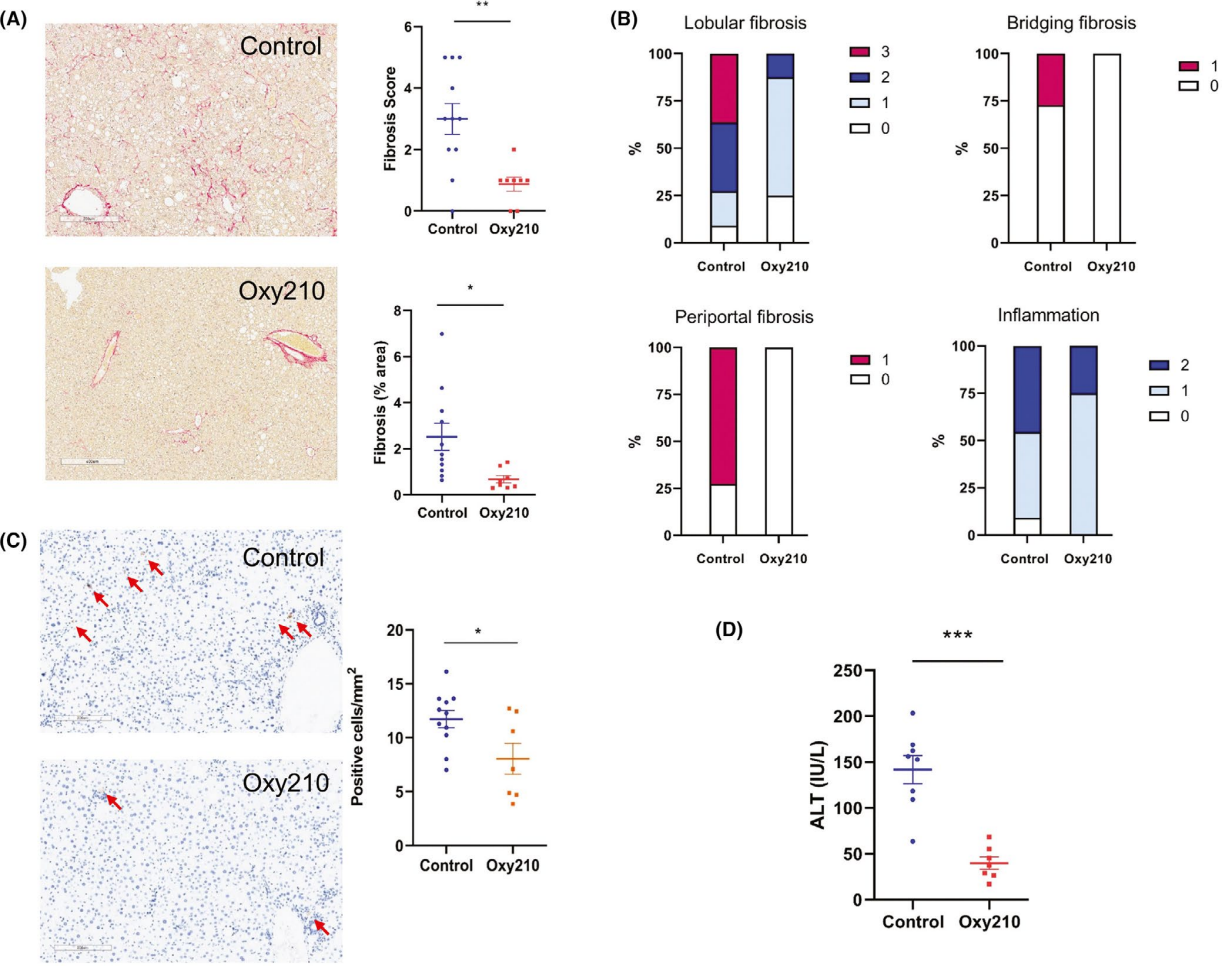
Changes in hepatic histology by Oxy210 treatment. Liver sections from control and Oxy210‐fed mice (n = 8–11 animals per group) were stained for collagen with picrosirius red (A). The percentage of fibrosis area in the entire liver sections was quantified by a computer algorithm^14^ (upper panel). Fibrosis score was determined by a pathologist blinded to the study (lower panel). (B) Incidence of lobular fibrosis, bridging fibrosis, periportal fibrosis and inflammation is presented as a percentage of mice in each group exhibiting these pathologies. Pathologist's scoring criteria are listed in Table S2. (C) Apoptotic cells detected by immunohistochemical analysis. Positive cleaved caspase 3 staining (brown) are indicated by red arrows. Nuclei were detected by DAPI staining (blue). (D) Plasma ALT were measured by colorimetric assay. Results are presented as mean ± SEM. *denotes *p* < 0.05, **denotes *p* < 0.01 and ***denotes *p* < 0.01 versus control

**FIGURE 5:**
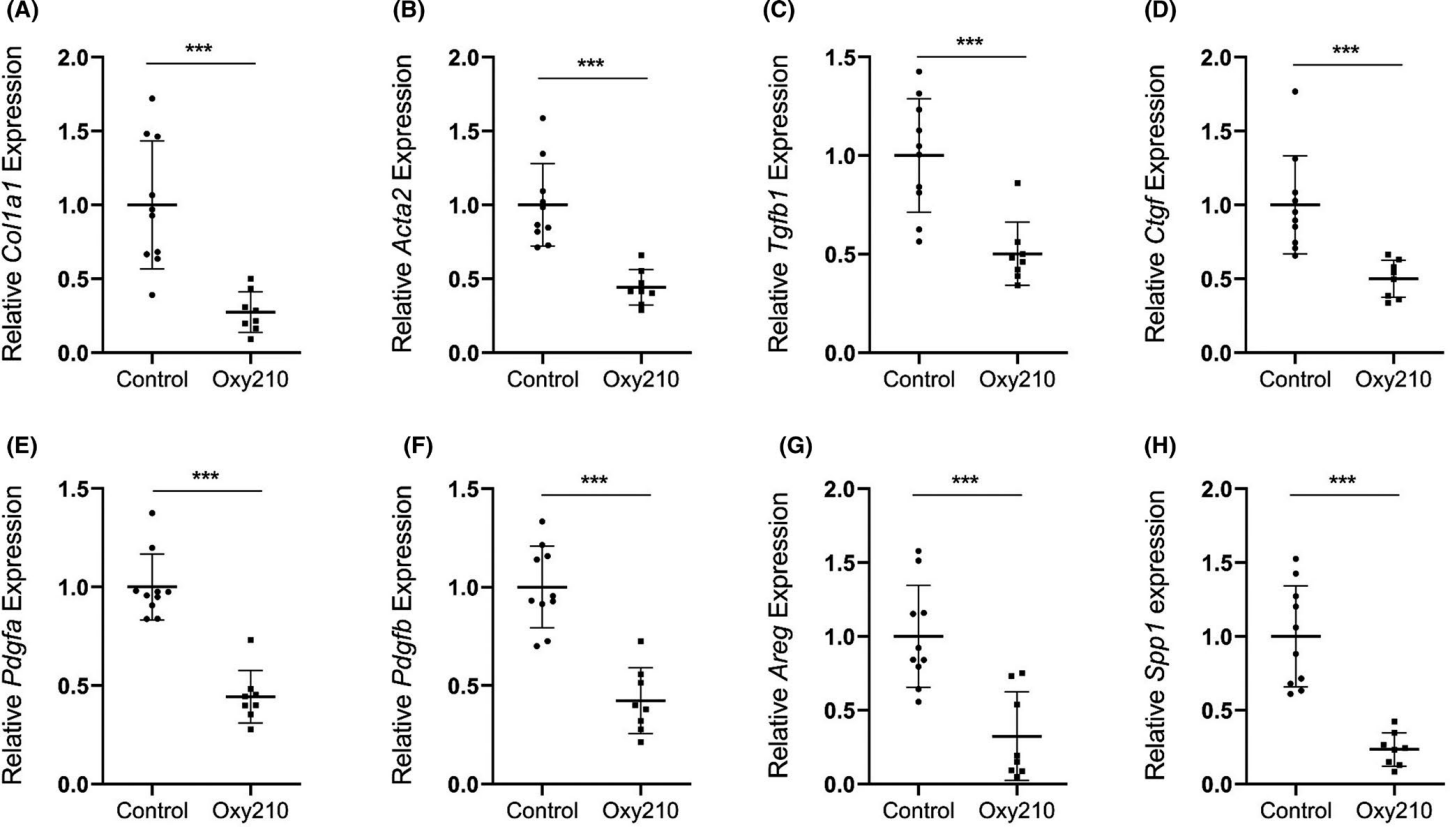
Effects of Oxy210 on hepatic gene expression. Expression of pro‐fibrotic genes *Col1a1* (A), *Acta2* (B), *Tgfb1* (C), *Ctgf* (D), *Pdgfa* (E), *Pdgfb* (F), *Areg* (G), and *Spp1* (H) in the livers from control and Oxy210‐fed mice (8–10 mice in each group) was measured by qPCR and normalized to the level of the housekeeping gene *Oaz1*. Relative gene expression levels are presented as mean ± SD. ***denotes *p* < 0.001 versus control

We assessed the suitability of Oxy210 as an orally delivered drug candidate. As the liver is the primary target organ in NASH, limiting systemic exposure of the drug candidate may be desirable to minimize potential side effects outside of the liver. Mice were fed Oxy210‐supplemented Western diet (WD) for 96 h. As shown in Figure 2F ^,^ after oral absorption when administered in food, significant quantities of Oxy210 reside in the liver (4.53 ± 1.1 µM equivalent of 1905 ± 466 ng/ml) and the liver concentration of Oxy210 was about 8.4‐fold higher than that in the plasma (0.53 ± 0.29 µM equivalent of 226 ± 123 ng/ml). The plasma concentration achieved through administration in food was lower compared to the peak plasma concentration observed after oral gavage,^30^ suggesting that oral absorption of Oxy210 is somewhat diminished in the presence of food. To examine the potential disease‐modifying effects of Oxy210 *in vivo*, we tested whether treatment with Oxy210 would attenuate liver fibrosis in mice. We previously reported that transgenic mice (CL‐Tg mice) carrying both the human APOE*3‐Leiden and CETP transgenes developed NASH and fibrosis after a 16 week of feeding a high‐fat high‐cholesterol diet. Unlike the choline‐deficient diet model,^20^ NASH in these mice is associated with hyperlipidemia, obesity and insulin resistance, features commonly seen in human NASH patients.^14^ Additionally, these mice recapitulate salient histological features of human NASH.^14^ Comparison of liver transcriptomics and network analyses showed a high resemblance to that of human NASH livers.^14^ Among >100 inbred mouse strains, we observed that CL‐Tg mice in the (C57BL/6J x BXD19/TyJ) F1 background developed the highest diet‐induced fibrosis and represent the most robust model for NASH phenotypes.^14^ In the present study, we tested the efficacy of Oxy210 in this model of NASH that has high relevance to human pathology. Female CL‐Tg mice in (C57BL/6 x BXD19/TyJ) F1 background were fed with a diet supplemented with Oxy210 at 4 mg of Oxy210 per gram of chow diet or 0.4% (wt/wt) whereas control mice were fed the same diet without Oxy210 supplement (Figure 3A). No significant difference in food intake was observed between control (1.96 ± 0.12 g/mouse/day) and Oxy210 (1.93 ± 0.20 g/mouse/day) groups. This actual rate of consumption resulted in the Oxy210 delivery dose of about 260 mg/kg/day. No overt signs of toxicity, such as changes in grooming behaviour or other vital activities, throughout the study period were observed in Oxy210‐treated mice and no overt changes in organ size and appearance were observed upon necropsy of the animals at the end of the study. Compared to the control animals, animals fed the Oxy210 diet for 16 weeks had a small but significant reduction in weight gain (Figure S2A, B) but no significant differences in adiposity, % liver weight and % gonadal fat weight were observed (Figure S2C‐E). Although the reasons for the body weight gain difference and its significance are presently unknown, given the fact that weight gain plays a detrimental role in the pathogenesis of NAFLD, and weight loss improves the condition, Oxy210‐induced reduction in weight gain may be an attractive feature of Oxy210.^37^ Hepatic lipid analysis showed that unesterified cholesterol was decreased in Oxy210‐fed mice whereas triglyceride, total cholesterol, cholesterol ester and phospholipid levels were not significantly different from that of control mice (Figure 3B‐F). Hepatic expression of LDL receptor (*Ldlr*, Figure S3A) and genes in cholesterol biosynthesis pathway (*Hmgcr*, *Fdft1*, *Dhcf7*, *Sqle*, *Srebf1* and *Srebf2*) was not decreased in Oxy210‐fed animals (Figure S3B‐G). Genes involved in cholesterol esterification (*Lcat*) and catabolism (*Cyp7a1*) were also not affected by Oxy210 treatment. We assessed the amount of pathological collagen in picosirius red stained liver sections and quantified by a computer algorithm, which we have previously shown to be more sensitive than the biochemical assay of hydroxyproline.^14^ Compared to the livers of control mice, Oxy210‐fed mice showed significant reduction in hepatic fibrosis with ~73% decrease in collagen‐positive stained area (Figure 4A, top right panel). Blinded assessment by a pathologist also showed a significant reduction in fibrosis score in Oxy210‐fed mice (Figure 4A, bottom right panel) with improvements in lobular fibrosis, bridging fibrosis, periportal fibrosis and inflammation (Figure 4B). Cleaved caspase 3 staining of liver sections showed a significant reduction in the number of apoptotic cells in Oxy210‐treated mice (Figure 4C). Consistent with this finding, plasma level of ALT, an indicator of liver damage, was significantly reduced in Oxy210‐treated mice (Figure 4D), indicating that Oxy210‐treated mice have lesser liver injury and that Oxy210 did not cause liver toxicity. These data show that Oxy210 treatment protects against diet‐induced liver injury and cell death in the liver, and attenuates diet‐induced liver fibrosis in vivo. In line with decreased liver fibrosis and injury in Oxy210‐fed mice, hepatic expression of collagen (*Col1a1*) and α‐smooth muscle actin (*Acta2)* were substantially reduced (Figure 5A,B). Expression of pro‐fibrotic cytokines (*Tgfb1*, *Ctgf*, *Pdgfa* and *Pdgfb*) was also significantly reduced in Oxy210‐fed mice (Figure 5C‐F). Oxy210 treatment also dramatically decreased the expression of the HSC activation markers, *Areg* and *Spp1* (Figure 5G‐H). Taken together, feeding of Oxy210 suppressed diet‐induced hepatic fibrosis in mice.

**FIGURE 6:**
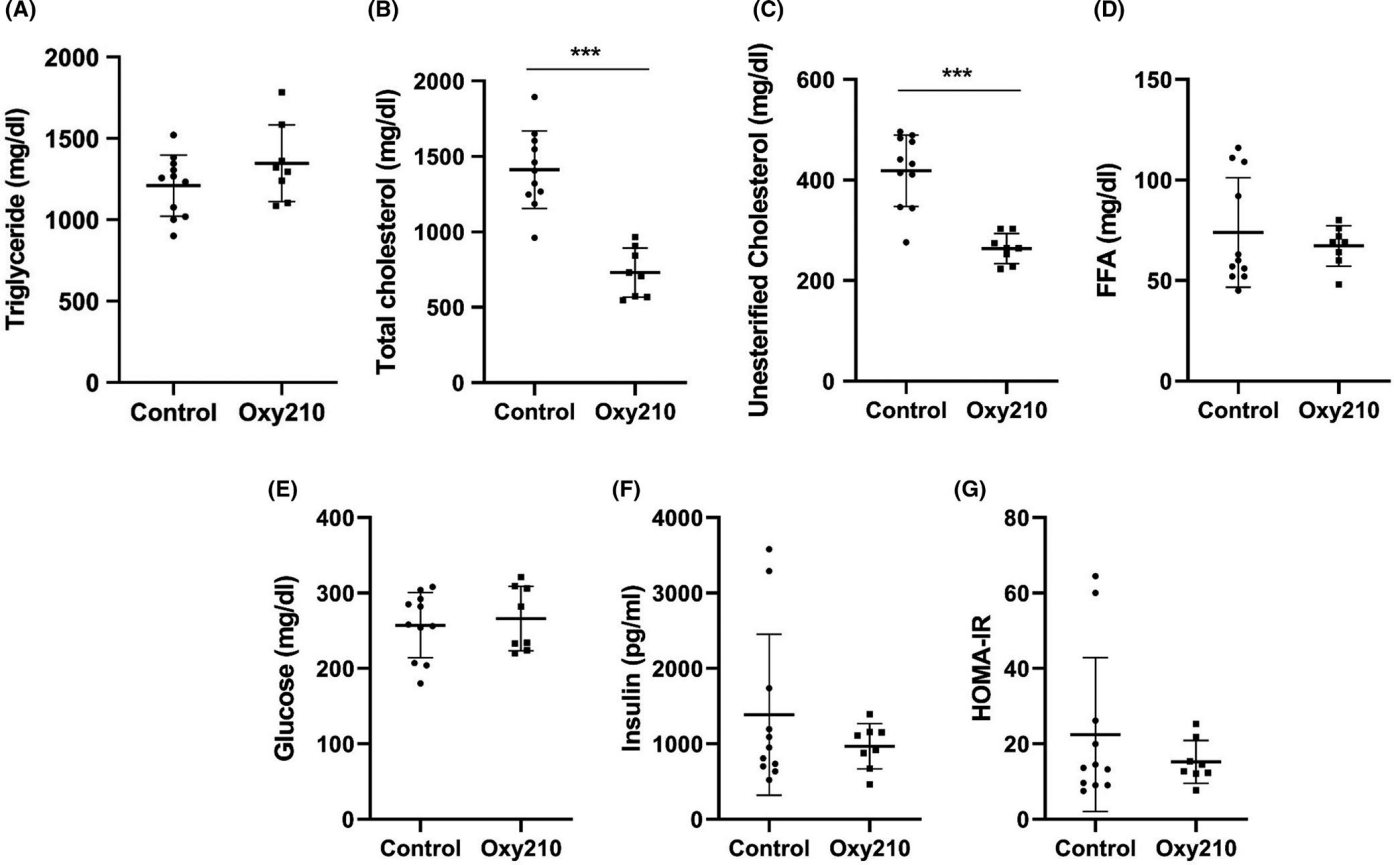
Effects of Oxy210 on plasma lipid and glucose metabolism. Control and Oxy210‐fed mice were fasted for 4 hrs. Plasma triglyceride (A), total cholesterol (B), unesterified cholesterol (C), free fatty acids (FFA, D) and glucose (E) were measured by colorimetric assays. Insulin levels (F) were measured by ELISA and calculated HOMA‐IR are presented in (G). Data are presented as mean ± SD (n = 8–11 mice per group). ***denotes *p* < 0.001 versus control

As in human NASH, hepatic fibrosis is often associated with dyslipidemia. Plasma total cholesterol and unesterified cholesterol levels but not triglyceride levels were significantly lower in Oxy210‐fed mice (Figure 6A‐C). No differences in plasma free fatty acids, glucose, insulin and insulin resistance (as assessed by HOMA‐IR) were observed (Figure 6D‐G).

**FIGURE 7:**
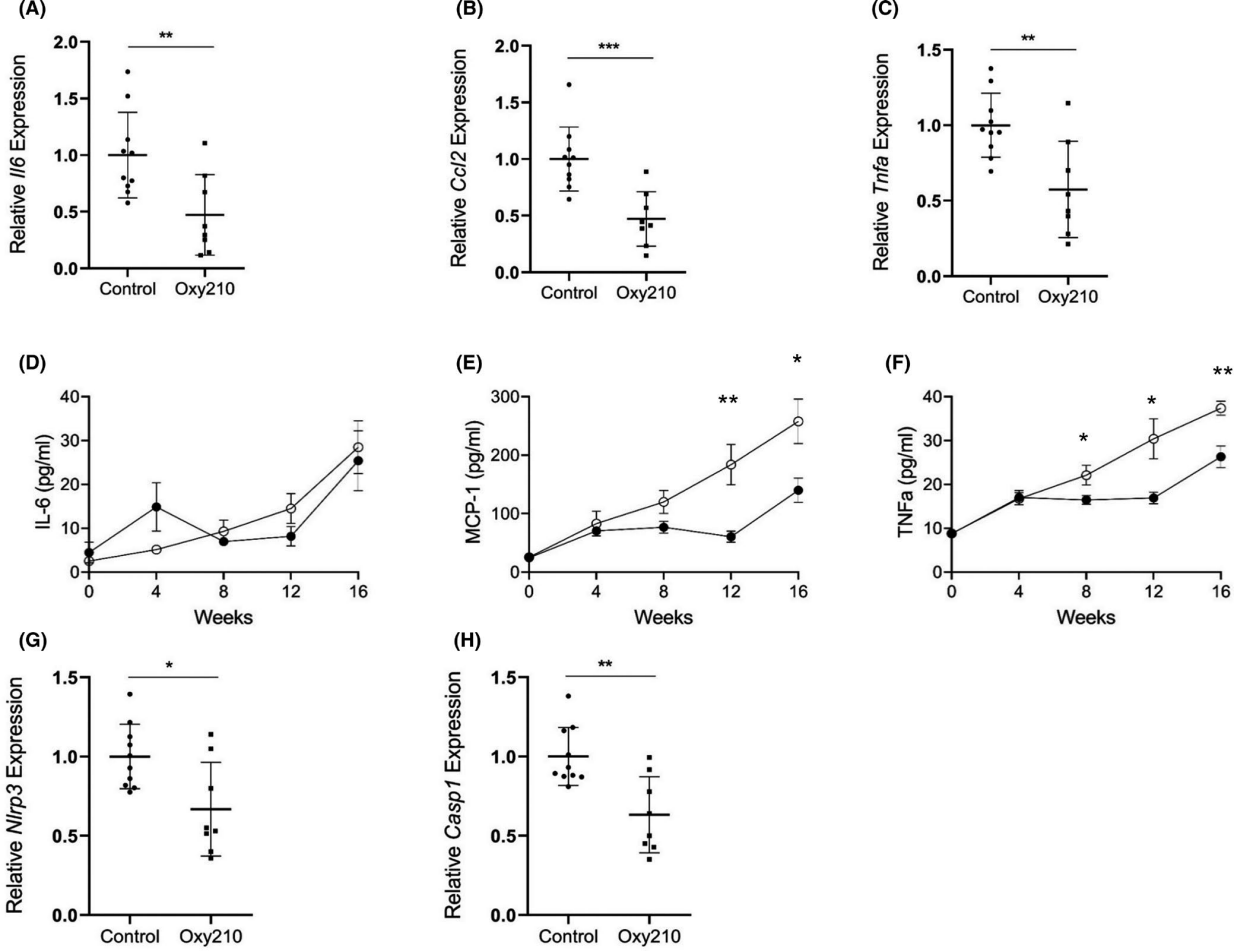
Effects of Oxy210 on pro‐inflammatory cytokines and inflammasome gene expression. Expression of pro‐inflammatory genes, *Il6*(A), and MCP‐1 (official gene name *Ccl2*, B) and *Tnfa* (C) in the livers from control and Oxy210‐fed mice (8–10 mice in each group) was measured by qPCR and normalized to the level of the housekeeping gene *Oaz1*. Relative gene expression levels are presented as mean ±SD. Plasma levels of IL‐6 (D), MCP‐1 (E) and TNF‐α (F) were measured by ELISA. Data are mean ± SEM from 8 mice per group. Liver expression of *Nlrp3* (G) and *Casp1* (H) was measured by quantitative PCR and normalized to *Oaz1*. Data are presented as mean ± SD from 8 to 10 mice per group. *denotes *p* < 0.05, ** denotes *p *< 0.01 and ***denotes *p* < 0.001 versus control

Inflammation plays an important role in the pathogenesis of NASH.^9^ Previously, we showed that progression of liver fibrosis correlated with plasma pro‐inflammatory cytokine levels and gene expression in the liver livers of ApoE*3‐Leiden.CETP mice.^14^ Compared to control mice, hepatic expression of *Il6* (interleukin‐6), *Ccl2* (MCP‐1) and *Tnfa* were significantly decreased in mice fed Oxy210 (Figure 7A‐C). We followed plasma levels of these cytokines during the 16‐week feeding of the high‐fat high‐cholesterol diet. In the control mice, plasma levels of IL‐6, MCP‐1 and TNFα increased during the progression of NASH (Figure 7D‐F). In Oxy210‐fed mice, the increase in MCP‐1 and TNFα levels was diminished whereas the levels of IL‐6 were not significantly different from the control mice (Figure 7D‐F). These data suggest that Oxy210 suppresses inflammation in vivo. Consistent with this finding, hepatic expression of inflammasome genes (*Nlrp3* and *Casp1*) was reduced in Oxy210‐fed mice (Figure 7G&H).

### 3.3 Oxy210 displays a favourable safety profile

SMO antagonists, a class of Hh pathway inhibitors that have been approved by the FDA for treatment of cancers that arise from aberrant Hh signalling as a result of alternations in SMO or elements upstream of SMO (ie basal cell carcinoma and acute myeloid leukaemia) have been reported to have teratogenic effects and foetal toxicity.^25^ In order to examine potential teratogenic effects of Oxy210 on foetus development, we administered Oxy210 at doses of 0.3mg/g to 10mg/g formulated in food to pregnant CD1 mice from gestation day 6 (GD6) to GD16 and assessed the health of the foetuses on GD17. These doses of Oxy210 in chow diet fed to pregnant CD1 mice resulted in actual doses of 44 mg/kg/day to 1,525 mg/kg/day based on food consumption. CD1 mice are FDA qualified rodent model for reproductive toxicity studies of investigational drugs. Visceral and skeletal examination of all foetuses at all doses of Oxy210, which inhibits Hh signalling not through SMO antagonism but downstream of SMO, epistatic to Sufu and at the level of Gli transcription activity,^30^ showed no signs of adverse effects. These data indicate that in contrast to SMO antagonists, Oxy210 is safe and does not cause reproductive developmental harm in mice.

## 4 DISCUSSION

We report an in vivo study of the effects of Oxy210, an oxysterol inhibitor of Hh and TGF‐β signalling, on hepatic fibrosis using a mouse model that displays many of the features and molecular signatures characteristic of human NASH pathophysiology.^14^ We demonstrate that oral delivery of Oxy210 in food resulted in significant liver exposure measured after 96 h (liver to plasma ratio: 8, Figure 2B) and dramatically reduced hepatic fibrosis in mice over the course of the 16‐week study (Figure 4). Additionally, we observed several beneficial effects of Oxy210 treatment related to NASH phenotypes: (a) pro‐inflammatory cytokine gene expression levels in the liver and plasma pro‐inflammatory cytokine levels were significantly reduced, (b) pro‐fibrotic cytokine and inflammasome gene expression in the liver was reduced; (c) apoptosis of liver cells and liver damage were reduced; (d) while hepatic triglyceride and total cholesterol contents were not significantly different from control mice, hepatic unesterified cholesterol accumulation was reduced; and (e) plasma total and unesterified cholesterol levels were significantly reduced. Together, these data support the concept that Oxy210 administration attenuates NASH and hepatic fibrosis in vivo, suggesting Oxy210 may be a potential pharmacological means to intervene in NASH.

Oxy210, an oxysterol‐based drug candidate, represents a novel class of small molecules for NASH therapy. As a structural class, the sterol space in general and oxysterols in particular have been underexplored and understudied as a source of small molecule drug candidates with desirable safety profile and favourable absorption, distribution, metabolism and excretion (ADME) properties. MAX BioPharma has identified a series of oxysterol analogues for development as novel therapies for various human disorders including cancer, bone healing, viral infections and fibrosis. In our experience, oxysterol‐based drug candidates are often relatively benign, orally bioavailable, nontoxic substances, unlikely to induce immune reactions and inexpensive to synthesize on large scale. Interestingly, unlike other oxysterol Hh pathway antagonists in our portfolio, Oxy210 antagonizes both Hh and TGF‐β signalling in fibroblasts and in primary human HSC. To our knowledge, this is the first evidence of efficacy of dual inhibition of Hh and TGF‐β signalling for inhibiting NASH. Unlike other FDA approved Hh pathway antagonists (Vismodegib, Sonidegib and Glasdegib) that bind to and inhibit SMO, inhibition of Hh signalling by Oxy210 occurs downstream of SMO receptor and epistatic to Sufu and at the level of *Gli* transcriptional activity.^24^, ^32^, ^33^ Inhibition of TGF‐β signalling by Oxy210 occurs at the level of SMAD2/3 phosphorylation and independent of inhibition of TGF‐β receptors I and II kinase activity, likely through a novel mechanism unlike that of other TGF‐β pathway inhibitors that are currently under clinical development in a number of pharmaceutical companies.^5^, ^34^ It is noteworthy that neutralizing antibodies to TGF‐β were reported to have adverse effects, including vascular and kidney inflammation, in preclinical mouse models of atherosclerosis and kidney disease.^21^ ^,^ ^23^ It is evident from our 16‐week NASH study that Oxy210 does not induce similar adverse effects, most likely due to a more selective inhibition of TGF‐β signalling yet to be elucidated.

In NASH, liver fibrosis develops following sustained injury to hepatocytes and organ inflammation. HSCs are the primary source of activated myofibroblasts that drive the fibrotic process.^22^ Activation and trans‐differentiation of quiescent HSCs play a key role in liver fibrosis.^12^ HSCs stimulated by TGF‐β and Hh ligands undergo epithelial‐to‐mesenchymal transition (EMT) into myofibroblasts, which exhibit a pro‐fibrotic phenotype. The reduction in hepatic fibrosis in Oxy210‐fed mice is consistent with our in vitro data showing that Oxy210 inhibits TGF‐β and Hh signalling in cultured primary HSCs (Figure 2). In Oxy210‐fed mice, pro‐fibrotic cytokines (TGF‐β, PDGF and CTGF) were all substantially reduced when compared to mice on control diet. The reduction in TGF‐β levels is likely to further enhance the inhibitory effects of Oxy210 in dampening the positive feedback loop of fibrosis mediated by TGF‐β. Given the role of TGF‐β and Hh signalling in fibrogenesis, Oxy210 has the potential to be a drug candidate for fibrotic diseases of non‐NASH aetiology and/or other organ systems (such as pulmonary and renal fibrosis). It would be interesting to examine the efficacy of Oxy210 in treating fibrosis in animal models of other human fibrotic diseases.

Besides its direct effects on TGF‐β and Hh signalling in fibrosis, it is interesting to note that cholesterol metabolism was altered in Oxy210‐fed mice. Plasma cholesterol (total and unesterified) and hepatic unesterified cholesterol levels were significantly reduced in Oxy210‐treated animals. It is unknown how Oxy210 affects cholesterol homeostasis in the liver. Since the diet used in this study contains large amount of cholesterol, the demand for *de novo* cholesterol biosynthesis is low and hence the effect of Oxy210 on *de novo* cholesterol biosynthesis is likely to be minimal in these animals. Consistent with this notion, hepatic gene expression of LDLR and key cholesterol metabolism enzymes were not decreased in Oxy210‐treated mice (Figure S3). Moreover, Oxy210 is unlikely to cause inhibition of cholesterol absorption in the gut since total hepatic cholesterol levels were similar in control vs. Oxy210‐treated mice. The improvement of fibrosis in these mice is consistent with our previous finding that liver fibrosis significantly correlated with plasma cholesterol and hepatic unesterified cholesterol.^14^ Time course studies also showed that onset of fibrosis coincided with a sharp increase in hepatic unesterified cholesterol levels.^14^

The present study shows that even with long‐term exposure to dietary Oxy210 at a dose that clearly ameliorated liver pathology, Oxy210 does did not cause any observable toxic effects in the mice. In fact, liver function was notably improved in the Oxy210‐treated mice, illustrated by the lower plasma ALT levels (Figure 4D). The favourable bioavailability profile combined with a high liver‐to‐plasma exposure ratio (Figure 2F) may suggest that systemic side effects exerted by Oxy210, if any, could be manageable. Future studies in animals with more advanced NASH (cirrhotic animals and possibly animals with hepatic decompensation) will be carried out to better characterize the metabolism and pharmacokinetics of Oxy210. Beyond this particular study, we have accumulated additional evidence suggesting Oxy210 has a favourable safety profile, such as the absence of significant in vitro hERG activity (data not shown) and a lack of teratogenic effects during an in vivo study in pregnant mice (data not shown). Teratogenicity, unfortunately, is a common undesirable feature of many Hh pathway antagonists that bind to and inhibit SMO, including Vismodegib,^26^ which carries a ‘black box warning’, according to the FDA. Although generally skewed towards an older patient population, a significant minority of NASH patients may be women of childbearing age, and thus, this issue deserves careful consideration and study.

In conclusion, we demonstrated that Oxy210 treatment can effectively ameliorate hepatic fibrosis and reduce hypercholesterolemia in mice. Our findings suggest that oxysterol derivatives, such as Oxy210, are a new class of drug candidates to be considered for NASH therapy.

## CONFLICT OF INTEREST

F.W, F.S. and F.P. have financial interest in MAX BioPharma and its intellectual property.

## AUTHOR’S CONTRIBUTION

STH, FW and FS contributed to the acquisition, analysis and interpretation of the data and to the preparation and critical revision of the manuscript. SWF and CEM contributed to the acquisition, analysis and interpretation of the data. FP and AJL contributed to the study concept and design and to the preparation and critical revision of the manuscript.

## Supporting information

Figure S1

Figure S2

Figure S3

Table S1

Table S2

Supplementary Material

## Data Availability

The data that support the findings of this study are available from the corresponding author upon reasonable request.
