# Supplementary figures and images for: Oxy210, a novel inhibitor of hedgehog and TGF‐β signalling, ameliorates hepatic fibrosis and hypercholesterolemia in mice
Source: Endocrinol Diabetes Metab. 2021 Aug 31;4(4):e00296. doi: 10.1002/edm2.296 (PMC8502222; doi:10.1002/edm2.296)

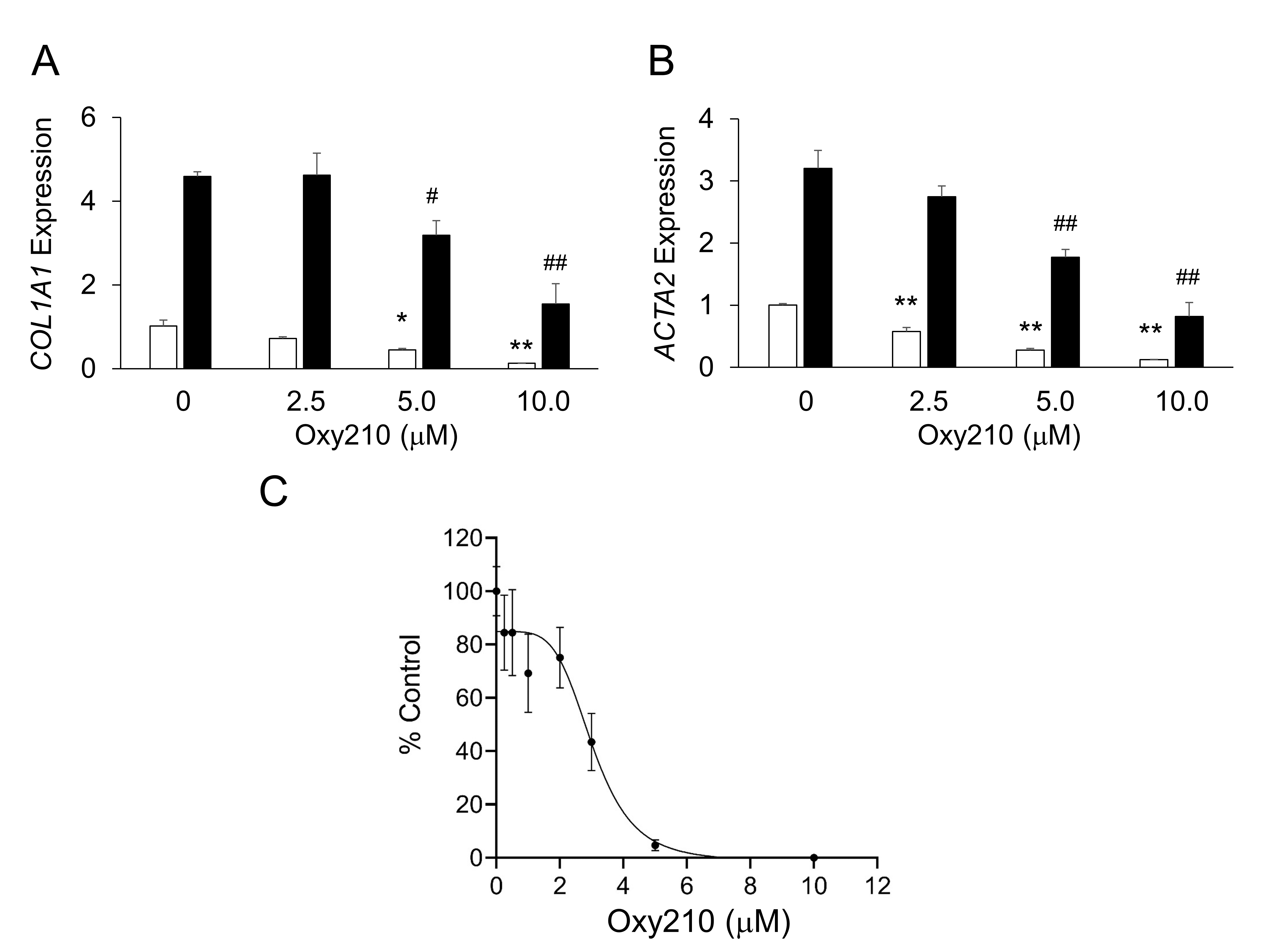

Supplement: Supplementary file 1 — Figure S1 [file EDM2-4-e00296-s001.PNG]

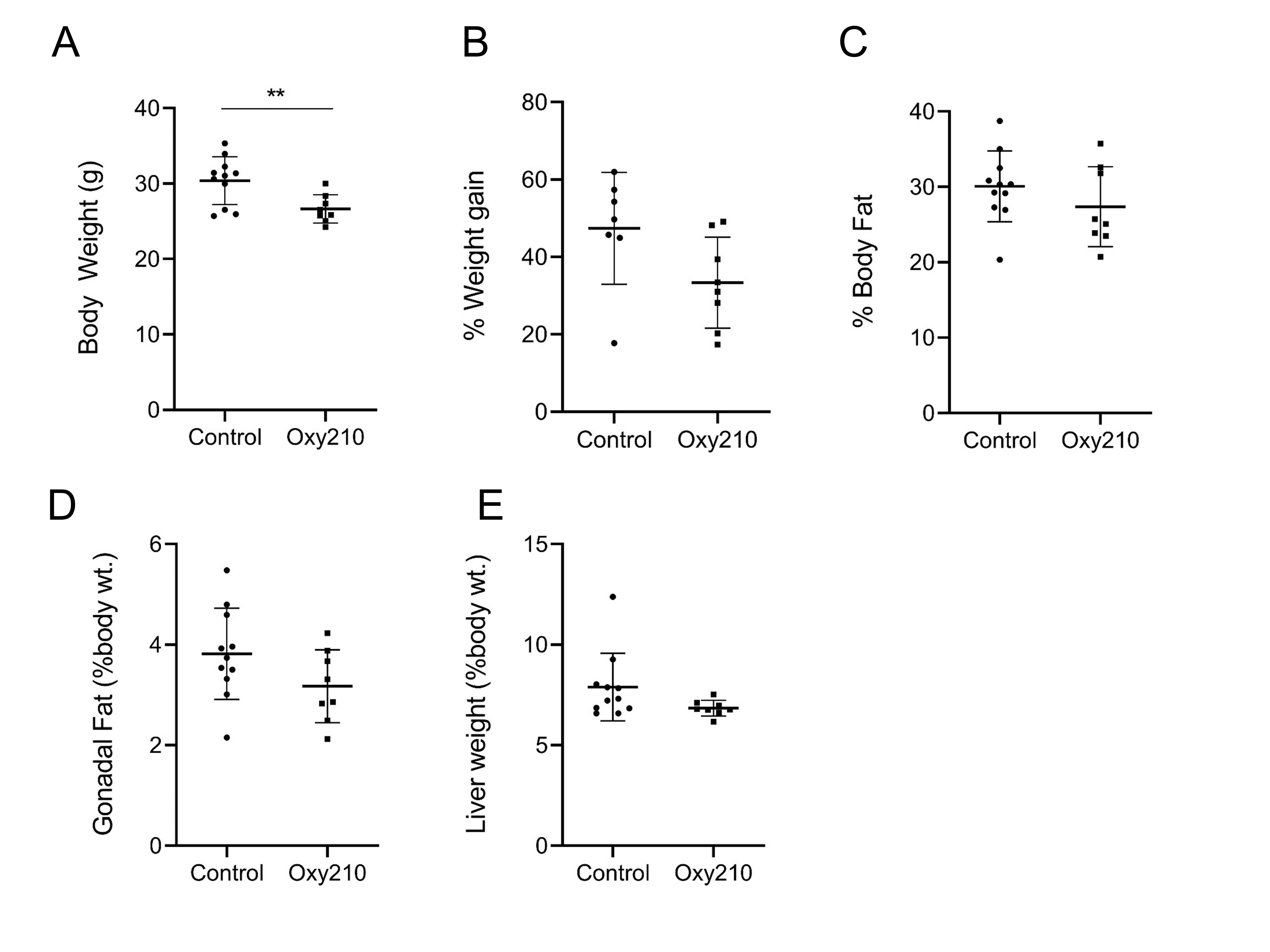

Supplement: Supplementary file 2 — Figure S2 [file EDM2-4-e00296-s003.PNG]

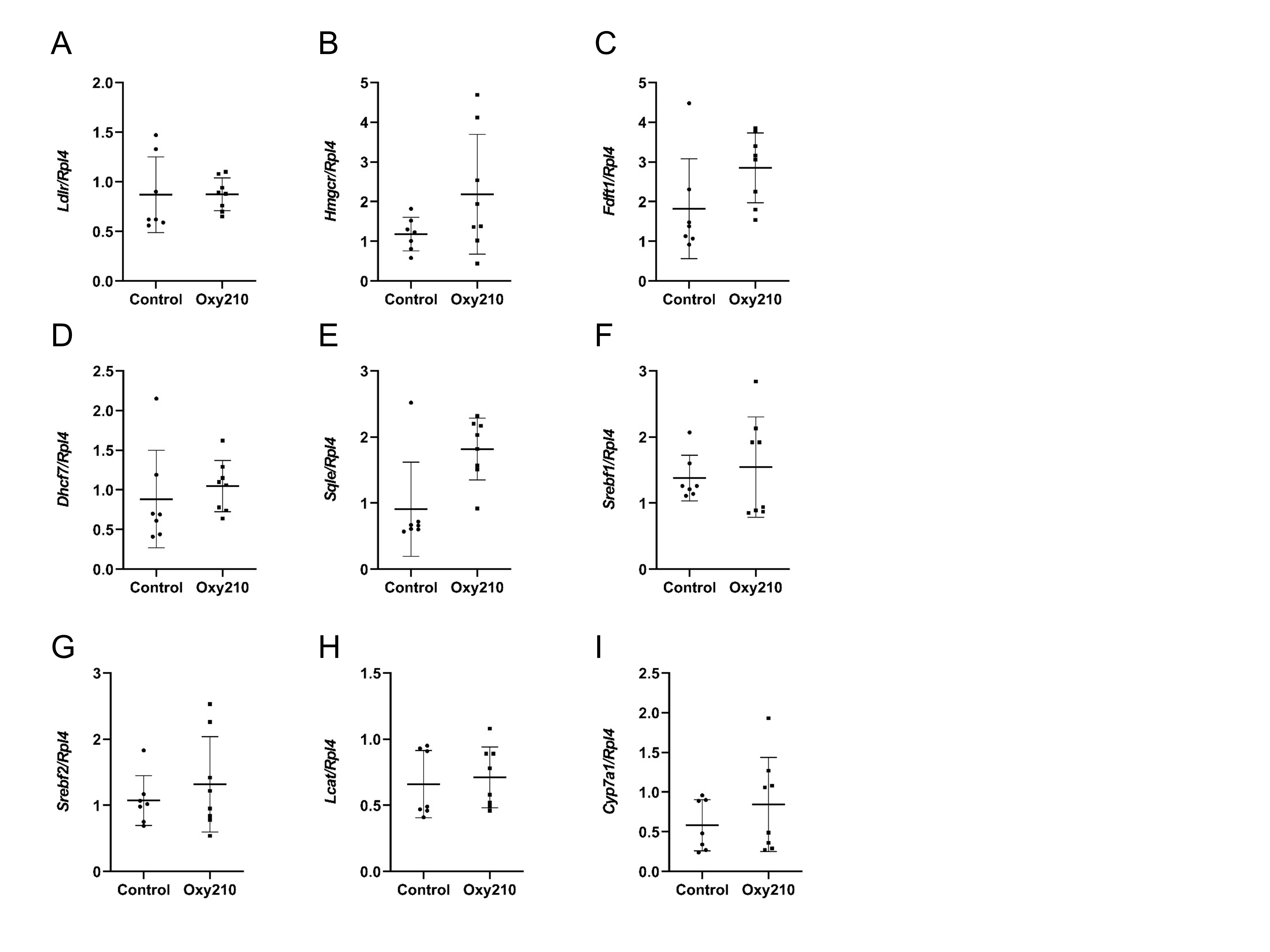

Supplement: Supplementary file 3 — Figure S3 [file EDM2-4-e00296-s002.PNG]
