# Supplementary material for: Oxy210, a novel inhibitor of hedgehog and TGF‐β signalling, ameliorates hepatic fibrosis and hypercholesterolemia in mice
Source: Endocrinol Diabetes Metab. 2021 Aug 31;4(4):e00296. doi: 10.1002/edm2.296 (PMC8502222; doi:10.1002/edm2.296)
Supplement: Supplementary file 4 — Table S1 [file EDM2-4-e00296-s006.docx]

**Supplementary Table 1.** Primer Sequence for qPCR

| Human genes | 5’ primer | 3’ primer |
| --- | --- | --- |
| *GAPDH* | 5’-CCTCAAGATCATCAGCAATGCCTCCT-3’ | 5’-GGTCATGAGTCCTTCCACGATACCAA-3’ |
| *COL1A1* | 5’- GTGCGATGACGTGATCTGTGA-3’ | 5’- CGGTGGTTTCTTGGTCGGT-3’ |
| *ACTA2* | 5’-GTGTTGCCCCTGAAGAGCAT-3’ | 5’- GCAGGACACCTTTTTGCAGATG-3’ |
| *CTGF* | 5’-CAGCATGGACGTTCGTCTG-3’ | 5’-AAC CACGGTTTGGTCCTTGG-3’ |
| *THBS1* | 5’-TGCTATCACAACGGAGTTCAGT-3’ | 5’- GCAGGACACCTTTTTGCAGATG-3’ |
| *GLI2* | 5’-GCCCTCACCTCCATCAATGC-3’ | 5’-ACTCACTGCTCTGCTTGTTCTG-3’ |
|  |  |  |
| Mouse genes | 5’ primer | 3’ primer |
| *Oaz1* | 5’-CCACTGCTTCGCCAGAGAG-3’ | 5’-CCCGGACCCAGGTTACTA-3’ |
| *Col1a1* | 5’-GCTCCTCTTAGGGGCCACT-3’ | 5’- CCACGTCTCACCATTGGGG-3’ |
| *Acta2* | 5’-GTCCCAGACATCAGGGAGTAA-3’ | 5’- TCGGATACTTCAGCGTCAGGA-3’ |
| *Tgfb1* | 5’-CTCCCGTGGCTTCTAGTGC-3’ | 5’- GCCTTAGTTTGGACAGGATCTG-3’ |
| *Ctgf* | 5’-GGGCCTCTTCTGCGATTTC-3’ | 5’-ATCCAGGCAAGTGCATTGGTA-3’ |
| *Pdgfa* | 5’-GAGGAAGCCGAGATACCCC-3’ | 5’-TGCTGT GGATCTGACTTCGAG-3’ |
| *Areg* | 5’- GGGGACTACGACTACTCAGAG-3’ | 5’-TCT TGGGCTTAATCACCTGTTC-3’ |
| *Spp1* | 5’-CCATCTCAGAAGCAGAATCTCC-3’ | 5’-ATCGTCATCATCGTCGTCC-3’ |
| *Il6* | 5’-TAGTCCTTCCTACCCCAATTTCC-3’ | 5’-TTGGTCCTT AGCCACTCCTTC-3’ |
| *Ccl2* | 5’-TTAAAAACCTGGATCGGAACCAA-3’ | 5’-GCATTAGCT TCAGATTTACGGGT-3’ |
| *Tnfa* | 5’-CAGGCGGTGCCTATGTCTC-3’ | 5’-CGATCACCC CGAAGTTCAGTAG-3’ |
| *Nlrp3* | 5’- ATCAACAGGCGAGACCTCTG-3’ | 5’-GTCCTCCTGGCATACCATAGA-3’ |
| *Casp1* | 5’-AATACAACCACTCGTACACGTC-3’ | 5’-AGC TCCAACCCTCGGAGAAA-3’ |
| *Il1b* | 5’-GCAACTGTTCCTGAACTCAACT-3’ | 5’-ATCTTTTGGGGTCCGTCAACT-3’ |
