# Supplementary material for: Oxy210, a novel inhibitor of hedgehog and TGF‐β signalling, ameliorates hepatic fibrosis and hypercholesterolemia in mice
Source: Endocrinol Diabetes Metab. 2021 Aug 31;4(4):e00296. doi: 10.1002/edm2.296 (PMC8502222; doi:10.1002/edm2.296)
Supplement: Supplementary file 5 — Table S2 [file EDM2-4-e00296-s005.docx]

**Supplementary Table 2.** Scoring criteria used by pathologist for assessing fibrosis and NASH phenotypes.

|  | 0 | 1 | 2 | 3 | 4 |  |
| --- | --- | --- | --- | --- | --- | --- |
| Lobular fibrosis | absent | focal | multifocal | diffuse | x |  |
| Bridging fibrosis | absent | <34% | <67% | >66% | bridging |  |
| Periportal fibrosis | absent | present | x | x | x |  |
| Inflammation 20X |  | <2 foci | 2 to 4 | >4 | x | based on NASH CRN |
