# Supplementary material for: Oxy210, a novel inhibitor of hedgehog and TGF‐β signalling, ameliorates hepatic fibrosis and hypercholesterolemia in mice
Source: Endocrinol Diabetes Metab. 2021 Aug 31;4(4):e00296. doi: 10.1002/edm2.296 (PMC8502222; doi:10.1002/edm2.296)
Supplement: Supplementary file 6 — Supplementary Material [file EDM2-4-e00296-s004.docx]

**Supplementary Figure 1. Effects of Oxy210 on TGF-β response and proliferation in cultured LX-2 HSC.** LX-2 cells were cultured in DMEM containing 0.1% FBS overnight and then pretreated with 5 or 10 µM Oxy210 as indicated for 2 hours. The cells were then treated with TGF-β (10ng/ml) (filled bars) or vehicle control (empty bars). Expression of COL1A1 (A), and ACTA2 (B) were analyzed by qPCR and normalized to GAPDH expression. Data are presented as mean ± SD from 3 replicates per group. # denotes p<0.05 vs. TGF-β treated control; ##p<0.01 vs. TGF-β treated control; *p<0.05 vs TGF-β untreated control; **p<0.01 vs TGF-β untreated control. (C) LX-2 cells were plated at 10% confluence and treated with Oxy210 at the concentrations indicated for 5 days. Cells were then trypsinized and the number of cells determined.

**Supplementary Figure 2. Effects of Oxy210 on body weight and composition.** Body weight (A), % body weight gain over 16 weeks of Western diet (B), body fat percentage as measured by NMR (C), gonadal fat weight % (D), and liver weight % (E) of mice on control diet and Oxy210-supplemented diet are presented as mean ± SD. ** denotes p <0.01 versus control.

**Supplementary Figure 3. Effects of Oxy210 on hepatic cholesterol metabolism gene expression.** Expression of cholesterol metabolism genes (as indicated) in the livers from control and Oxy210-fed mice (8-10 mice in each group) was measured by qPCR and normalized to the level of the housekeeping gene *Rpl4*. Relative gene expression levels are presented as mean ± SD.

**Supplementary Table 1. Sequence of qPCR primers.** Sequence of forward and reverse primers used in qPCR analysis are listed.

**Supplementary Table 2. Scoring criteria used by pathologist to assess fibrosis and NASH phenotype.**
